# Inter-annual variation patterns in the carbon footprint of farmland ecosystems in Guangdong Province, China

**DOI:** 10.1038/s41598-022-18425-z

**Published:** 2022-08-19

**Authors:** Qiao Guotong, Chen Fei, Wang Na, Zhang Dandan

**Affiliations:** 1 School of Economics and Management, Anhui University of Science and Technology, Huainan, 232001 Anhui China; 2 Anhui University of Science and Technology Academic Affairs Office and Innovation College, Huainan, 231001 Anhui China

**Keywords:** Ecology, Agroecology, Ecosystem ecology, Agroecology, Ecosystem ecology, Astronomy and astrophysics

## Abstract

Carbon sequestration in farmland ecosystems is an important link in the world carbon cycle and plays an important role in regional carbon reduction. Guangdong, a major industrial and economic province in China, was used as the study area, and the period 2001–2020 was taken as the study period. The carbon emissions, sequestration, and footprint of farmland ecosystems in Guangdong were estimated using carbon emission factors for agricultural inputs that are closer to the actual situation in China. The study showed that: (1) Carbon average emissions from farmland in Guangdong during the study period was 3.7624 million t a^−1^, with a balanced overall trend of change, and that nitrogen fertilize applications was the main factor contributing to carbon emissions. (2) The carbon sink capacity of Guangdong farmland ecosystems showed an overall decreasing trend of 10.32%, with an average annual carbon sink of 19.0363 million t a^−1^. Paddy and sugar cane cultivations were the main factor of carbon sink in farmland. (3) The average annual carbon footprint of Guangdong's farmland ecosystems was 531,100 ha a^−1^, which was in a carbon surplus. Carbon surplus and footprint showed a decreasing trend year by year. The paper results provide a theoretical basis for the formulation of carbon emission reduction policies and industrial restructuring in Guangdong and provinces with the same industrial structure.

## Introduction

Climate change has become an issue of concern in political and economic fields around the world, which CO_2_ is an important greenhouse gas causing global warming. According to the report “The State of Global Climate 2020” published by the World Meteorological Organization, the global average molar fraction of CO_2_ has exceeded 410 ppm in 2020 and the total carbon emissions of China in 2020 will be 11,680 million tons. Since 2006 when China became the largest CO_2_ emitter, China has been working hard to combat global climate change^1^. China announced at the 75th session of the UN General Assembly that it would take stronger policies and measures to reach peak carbon emissions by 2030 and also aims to achieve carbon neutrality by 2060. However, as the only major economy to achieve carbon emission growth by 2020^2^, China will face the problem of carbon reduction and development in achieving the above “dual carbon” target. Agriculture occupies a special position in climate change. Meanwhile, soil respiration and fertilizers, pesticides production in agricultural production activities directly or indirectly lead to carbon emissions^3^. Photosynthesis of crops absorb CO_2_ and makes farmland ecosystems have a strong carbon sequestration effect^4^, so farmland ecosystems have the dual characteristics of carbon emissions and absorption. The effective control of carbon emissions from agricultural production and the proper use of the carbon sequestration capacity of crops that in farmland ecosystems will help China to formulate appropriate policies to achieve the carbon peaking and carbon neutrality.

The “carbon footprint” is derived from the ecological footprint to measure the CO_2_ emissions directly or indirectly caused by a certain activity^5,6^. Where the carbon footprint of a farmland ecosystem is defined as the productive land area required for the carbon emissions from the production activities of the farmland to be absorbed^7^. Several researchers have studied the carbon sources and sequestrations of farmland ecosystems^8–12^, and carbon footprint estimation and research based on carbon flow changes in farmland ecosystems has become one of the important research directions in the academic field^11–16^. Ling et al.^11^ analysed the carbon footprint of farmland ecosystems in Shandong Province from 2002 to 2013 to investigate the carbon sequestration capacity of farmland ecosystems and the differences between the cities. Xu et al.^13^ estimated the carbon footprint of paddy production in five typical paddy production areas in China. She et al.^14^ analysed the carbon structure and carbon sequestration capacity of crops based on the carbon footprint of major crops in typical agricultural areas in China. Duan et al.^15^ estimated the carbon footprint of major crops and farmland production inputs from 1990 to 2009 in China. Gan et al.^17^ quantified the carbon footprint of wheat under different tillage patterns to explore the carbon reduction capacity of new tillage patterns. Liu et al.^18^ conducted a comprehensive review based on the carbon footprint changes of crops grown in semi-arid areas under seven tillage patterns.

The above-mentioned studies have matured the method of accounting for the “carbon footprint”. Since farmland ecosystems are open and influenced by human factors^8^, such as differences in tillage patterns^19^ and differences in the climate of the farmland^20^, all affect the carbon emissions and sequestrations of farmland ecosystems. Therefore, the calculation of carbon emissions and sequestrations of farmland ecosystems needs to be updated according to time and regional differences. With the introduction of China's “dual carbon” target by 2020, the direction and intensity of carbon reduction policies will need to be updated and adjusted in the future. Whereas the analysis of agricultural carbon emissions, sequestrations, and footprints in China can be used as a reference for the government to formulate carbon reduction policies and measures for a certain period of time.

As a pioneering region in China's reform and the introduction of foreign investment, Guangdong Province can be said to be in the national spotlight for its green economic transformation under the “dual carbon” target. According to the 2019 China Statistical Yearbook and the Guangdong Statistical Yearbook, Guangdong's arable land covers more than 1.9 million hectares, accounting for more than 10% of the administrative area of Guangdong and 2% of the country's arable land. So, Guangdong cannot ignore the carbon emissions from agricultural production and the carbon sequestration role played by farmland ecosystems in the formulation of carbon reduction policies. Although Guangdong is one of the leading provinces in China in terms of industrialization and urbanization, there are problems left over from the historical development of agriculture. Such as emphasizing industry over agriculture and development over conservation, which are typical of the difficulties encountered in the industrialization process in other provinces in China. It can be said that Guangdong, which is at the forefront of industrialization in China, can provide a reference for other provinces with similar industrial structures, in the formulation of green carbon emission reduction policies under the “double carbon” target. On 30 September 2021, the Guangdong Provincial People's Government issued a policy document on the 14th Five-Year Plan for Promoting the Modernization of Agriculture and Rural Areas in Guangdong. The document also pointed out that the most difficult task for the province to achieve the second 100-year goal lies in agriculture, and called for a focus on developing ecological agriculture. After the “dual carbon” target was proposed, Guangdong agriculture requires modernization and carbon emission reduction, which requires more detailed and comprehensive control of carbon emissions from agricultural production, giving full play to the carbon sequestration role of farmland ecosystems. Also modernizing agriculture, and achieving stable economic development.

Thus, based on the relevant data of Guangdong from 2001 to 2020, the paper using carbon emission factors for agricultural inputs that are closer to the actual situation in China, estimates the carbon emissions, sequestration, and footprint of farmland ecosystems in Guangdong during the 20-year period based on the accounting methods of existing studies. It also analyses the inter-annual variation patterns, with a view to providing a theoretical basis and reference for the formulation of carbon reduction policies and the optimization of industrial structure layout in Guangdong and similar industrial structure provinces under the “dual carbon” target.

## Methods

### Calculation of carbon emissions from farmland ecosystems

The main sources of carbon emissions from farmland ecosystems are: carbon emissions from the production and use of agricultural production use, mainly fertilizers, pesticides, and agricultural films, and from the use of agricultural machinery that consumes fossil fuels. The calculation is expressed as follows.

1 $$E = \sum {E_{i} = \sum {G_{i} \times \gamma_{i} } }$$

where $E$ (tC a^−1^) is the carbon emission of farmland ecosystem. $i$ is the various productive inputs of farmland. $G_{i}$ is the quantity value of each productive input, including the amount of nitrogen, phosphorus, and potassium chemical fertilizers and compound fertilizers, pesticides, agricultural films, agricultural diesel oil usage, total power of agricultural machinery, irrigation area and farmland cultivation area. The farmland cultivation area is based on the actual planted area of crops and irrigation area is based on the effective irrigation area. $\gamma_{i}$ is the input carbon emission factors (Table 1)^21–23^.

**Table 1:** Carbon emission factors for farmland ecosystems.

	Fertilizer (kgC t^−1^)				Pesticides (kgC kg^−1^)	Agricultural film (kgC kg^−1^)	Agricultural diesel (kgC kg^−1^)	Total power of agricultural machinery (kgC kW^−1^ h^−1^)	Agricultural irrigation (kgC ha^−1^)	Tillage (kgC ha^−1^)
	Nitrogen	Phosphate	Potash	Compound						
Coefficient	2116	636	180	380.97	4.93	5.18	0.5927	0.18	20.476	16.47

### Calculation of carbon sequestration in farmland ecosystems

The calculation of carbon sequestrations in farmland ecosystems is mainly based on the principle of carbon sequestration by photosynthesis of crops. The amount of carbon sequestered in farmland ecosystems can be estimated from the crop yields by the Eq. (2)

2 $$A = \sum {A_{j} } = \sum {C_{j} \cdot D_{j} \cdot (1 - W_{j} )/H_{j} }$$

where $A$ (tC a^−1^) is the amount of carbon sequestered by the farmland ecosystems. $A_{j}$ is the amount of carbon uptake required to synthesize a unit of dry matter by the crop $j$. $D_{j}$ is the economic yield of crop $j$. $W_{j}$ is the water content of the economic yield of crop $j$. $H_{j}$ is the economic coefficient of the crop $j$ and $C_{j}$ is the carbon uptake rate of crop $j$. Where $C_{j}$, $W_{j}$, and $H_{j}$ of the main crops are shown in Table 2^15,16^.

**Table 2:** Water content, carbon uptake and economic coefficients for economic yield of major crops.

Indicators	Paddy	Wheat	Corn	Legumes	Yams	Sugar cane	Peanuts	Canola	Tobacco leaf	Vegetables	Other food crops
$W_{j}$ (%)	12	12	13.5	13	13.3	50	10	10	12	90	12
$C_{j}$	0.414	0.485	0.471	0.450	0.423	0.450	0.450	0.450	0.450	0.450	0.450
$H_{j}$	0.45	0.40	0.40	0.34	0.70	0.50	0.43	0.25	0.55	1.00	0.40

### Calculation of the carbon footprint of farmland ecosystems

According to the relevant definitions^5,15,16^, the carbon footprint of farmland is defined as the amount of productive land area required to absorb direct or indirect CO_2_ emissions from fossil fuel combustion caused by farmland production inputs. It can be obtained by comparing the carbon emissions from each farmland input with the carbon absorption capacity per unit of farmland area. The carbon footprint of an farmland ecosystem ($CEF$, ha a^−1^) is then the ratio of the total carbon emissions from the farmland ecosystem ($E$, tC a^−1^) to the total carbon sequestered per unit area of farmland ($A/S$, tC ha^−1^ a^−1^, $S$ is the area of arable land). The calculation is as follows.

3 $$CEF = \frac{E}{A/S}$$

Arable land area ($S$) represents the ecological carrying capacity of farmland ecosystems. The carbon deficit of farmland is expressed as the carbon footprint is greater than the ecological carrying capacity. If the carbon footprint of the farmland is less than the ecological carrying capacity, then it is a carbon ecological surplus. And the size of the surplus ($CS$) is the difference between the area of arable land and the area of the carbon footprint (Eq. 4).

4 $$CS = S - CEF$$

## Results

### Analysis of carbon sources in Guangdong farmland ecosystems under the “dual carbon” target

#### Analysis of inter-annual variation in carbon emissions from farmland ecosystems in Guangdong

**Figure 1:**
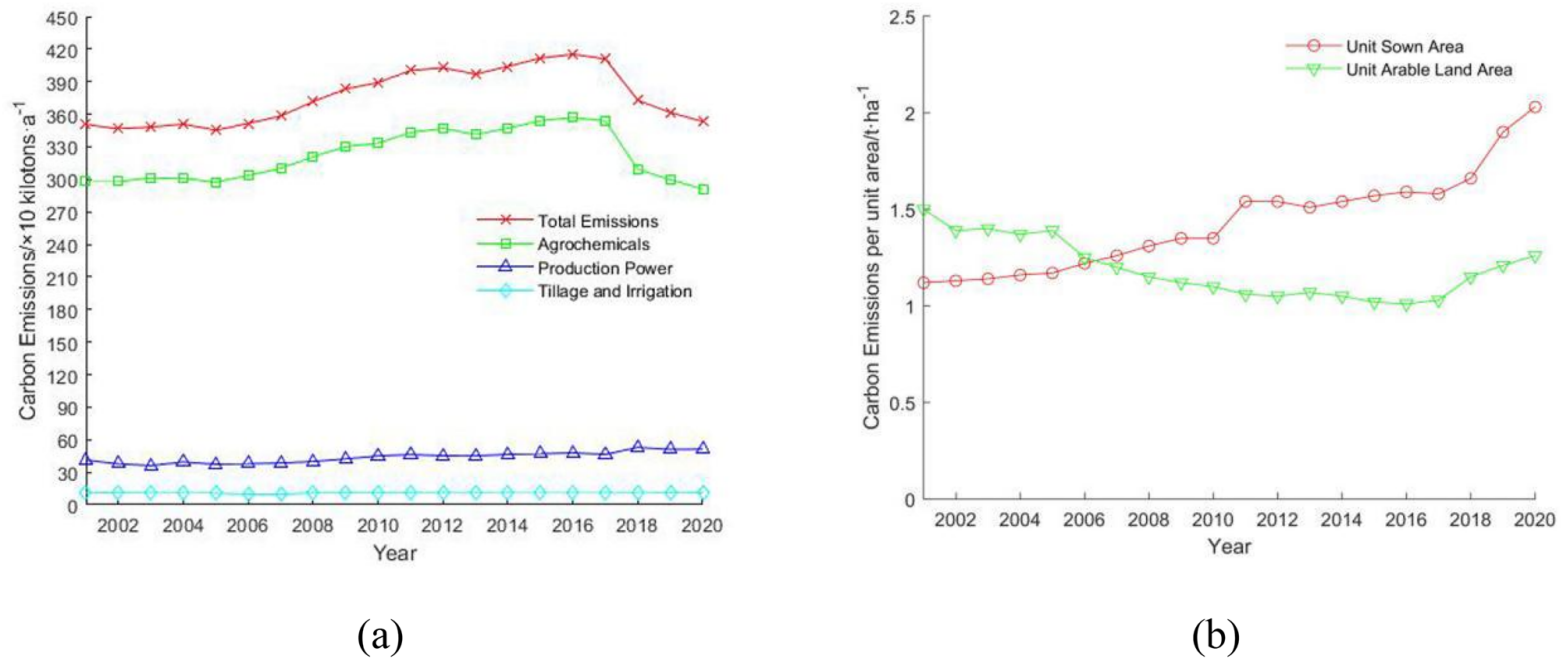
(**a**) Inter-annual variation of carbon emissions from farmland ecosystems in Guangdong; (**b**) inter-annual variation in carbon emissions per unit area of farmland ecosystems in Guangdong.

Guangdong's carbon emissions from farmland ecosystems showed an increasing trend year by year during 2001–2017 (Fig. 1a), with carbon emissions gradually reaching a peak of 4.153 million t a^−1^ in 2016 from 3.554 million t a^−1^ in 2001, but decreasing year by year from 2017 onwards. Eventually it's decreasing to 3.533 million t a^−1^ by 2020. Showing that Guangdong's farmland ecosystem carbon emissions have remained relatively flat over the past 20 years, with an average annual carbon emission of 3.7624 million t a^−1^. The carbon emissions per unit arable land area of Guangdong's farmland ecosystems show an increasing trend year by year (Fig. 1b), from 1.12 t ha^−1^ in 2001 to 2.03 t ha^−1^ in 2020, an increase of 81.25% over 20 years, with an average annual carbon emission per unit arable land area of 1.43 t ha^−1^. While the carbon emissions per unit sown area show the opposite trend to the total carbon emissions, from 2001 to 2016, showing a decreasing trend year by year. The carbon emissions per unit of sown area decreased from 1.50 t ha^−1^ in 2001 to 1.01 t ha^−1^ in 2016 and then started to increase year by year from 2017 to 1.26 t ha^−1^ in 2020, with an overall decrease of 16% and an average annual carbon emission per unit of sown area of 1.19 t ha^−1^.

#### Analysis of carbon sources in Guangdong farmland ecosystems

The carbon emissions from agricultural production power (estimated by the total power of agricultural diesel and agricultural machinery) in Guangdong's farmland ecosystems show an increasing trend year by year (Fig. 1a), from 411,000 t a^−1^ in 2001 to 513,000 t a^−1^ in 2020, an increase of nearly 25% in 20 years. Carbon emissions from tillage and irrigation inputs are relatively flat, from 116,000 t a^−1^ in 2001 to 109,000 t a^−1^ in 2020, with an average of 107,000 t a^−1^ over the last 20 years. Carbon emissions from chemicals in agricultural production (estimated by fertilizer, pesticide, and agricultural film inputs) have the greatest impact on the overall emissions, with carbon emissions from agricultural chemicals reaching 2.9097 million t a^−1^ in 2020, accounting for 82.36% of total carbon emissions from farmland ecosystems, but a relatively flat trend. Although the share of carbon emissions from agricultural production power is increasing year by year, the contribution of carbon emissions due to inputs of agricultural chemicals is still in an absolute position. The use of agricultural chemicals directly affects the carbon emissions of Guangdong's farmland ecosystems. Therefore, a more detailed analysis of the carbon emissions of various agricultural chemicals is necessary in order to make carbon reduction proposals.

**Figure 2:**
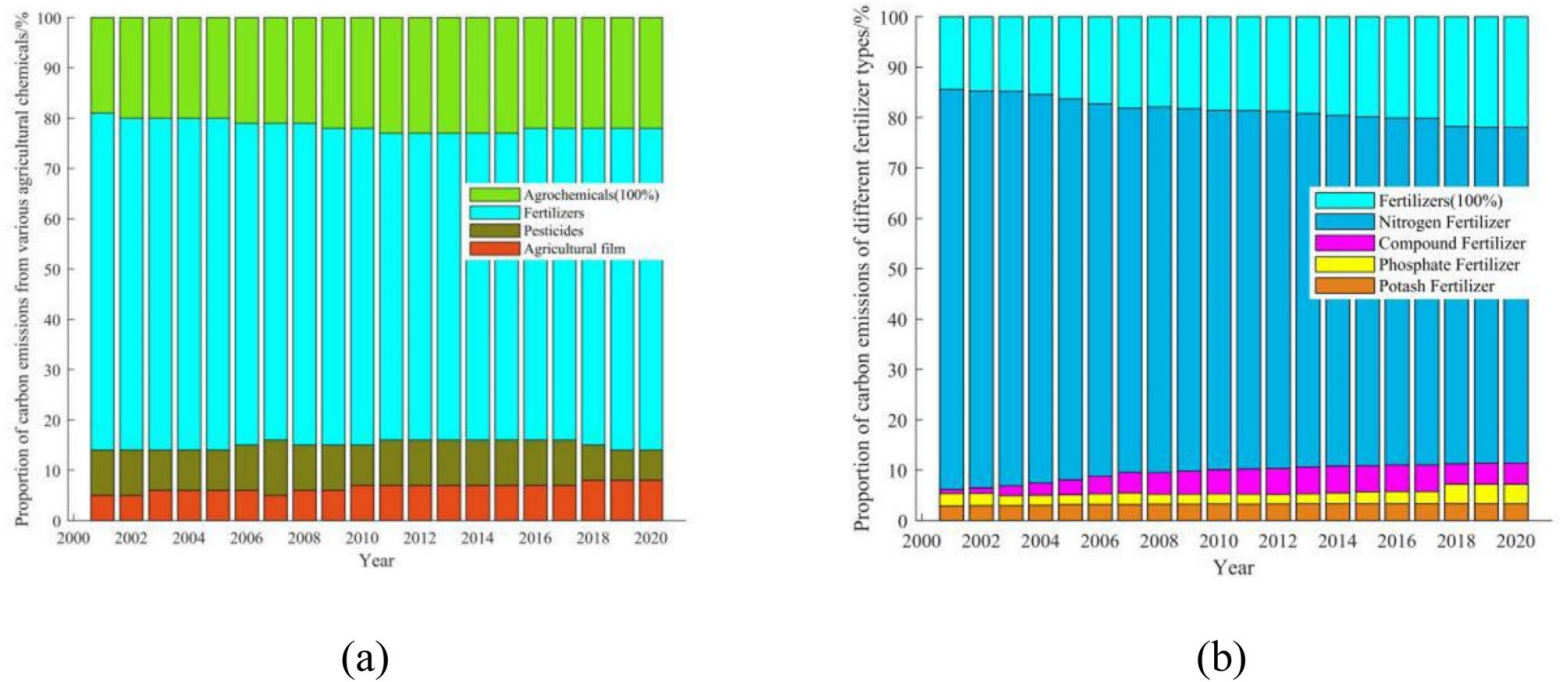
(**a**) Proportion of carbon emissions from various types of agricultural chemicals in Guangdong farmland ecosystems; (**b**) proportion of carbon emissions from different fertilizer types in Guangdong farmland ecosystems.

Depending on Fig. 2a, although the proportion of carbon emissions caused by agricultural films has been increasing year by year, chemical fertilizers still occupy an absolute position, with their carbon emissions accounting for 78.45% of agricultural chemicals on average in the past 20 years. Which the average proportions of carbon emissions caused by pesticides and agricultural films are 15.17% and 6.38% respectively. Among the carbon emissions from various fertilizers (Fig. 2b), the annual average share of carbon emissions in the past 20 years is distributed from the largest to the smallest: 81.63% from nitrogen fertilizers, 9.57% from compound fertilizers, 5.60% from phosphate fertilizers and 3.20% from potash fertilizers. From the trend of carbon emissions of various types of fertilizers, we can learn that the carbon emissions of nitrogen fertilizers have been decreasing year by year, from 85.63% in 2001 to 78.10% in 2020, and the emissions have slowly risen from 2.061 million t a^−1^ in 2001 to a peak of 2.1276 million t a^−1^ in 2016, then gradually decreased to 1.7797 million t a^−1^ in 2020. Compound fertilizers, on the other hand, rose from 6.17% in 2001 to 11.40% in 2020, an increase of nearly 85%, and their carbon emissions rose year by year from 148,600 t a^−1^ to a peak of 305,200 t a^−1^ in 2016 and then gradually fell to 259,900 t a^−1^ in 2020, an increase of 74.90%. The share of carbon emissions from potash is relatively stable, rising from 2.89% to 3.29%, reaching a peak of 91,800 t a^−1^ in 2016 and then gradually decreasing to 75,500 t a^−1^ in 2020. The share of carbon emissions from phosphate fertilizers is also on a year-on-year rise, from 5.31% to 7.20%, an increase of 37.47%. However, the carbon emissions from phosphate fertilizers do not produce a peak in 2016 but keep increasing in a relatively stable trend, with its carbon emissions rising from 127,800 t a^−1^ in 2001 to 164,100 t a^−1^ in 2020, an increase of 28.40%.

### Analysis of carbon sequestration in Guangdong farmland ecosystems under the “dual carbon” target

#### Analysis of inter-annual variation in the carbon sequestration function of Guangdong farmland ecosystems

**Figure 3:**
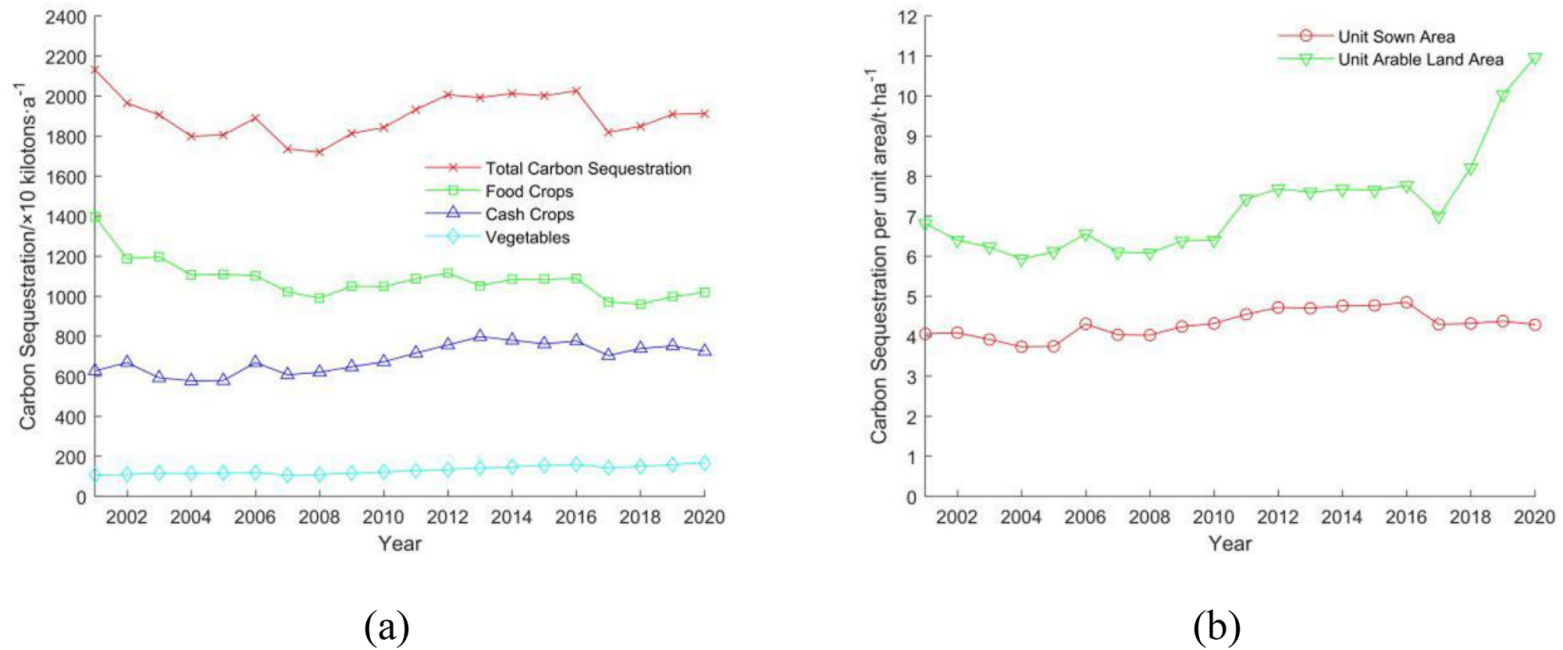
(**a**) Carbon sequestration function of farmland ecosystems in Guangdong; (**b**) inter-annual variation of carbon sequestration function per unit area of farmland ecosystems in Guangdong.

In the inter-annual variation of carbon sequestration function of farmland ecosystems in Guangdong (Fig. 3a), although there are fluctuations in the variation of total carbon sequestration in farmland ecosystems, the overall decrease is not significant. With the total carbon sequestration decreasing from 21.3176 million t a^−1^ in 2001 to 19.1178 million t a^−1^ in 2020, a decrease of 10.32% in the last 20 years, and the average annual carbon sequestration is 19.0363 million t a^−1^, among which the total carbon sequestration in 2008 is the lowest, only 17.2033 million t a^−1^.

The total carbon sequestered in 2008 was the lowest at 17.2033 million t a^−1^. The inter-annual variation of carbon sequestration by food crops (paddy, wheat, corn, legumes, yams, and other food crops) is similar to that of farmland ecosystems, decreasing from 13.9742 million t a^−1^ to 10.209 million t a^−1^, a decrease of 27%. The inter-annual variation of carbon sequestration by cash crops (sugarcane, peanuts, Canola, and tobacco) and vegetables generally shows a stable upward trend, with carbon sequestration increasing by 15.54% and 55.54% respectively over the past 20 years. Meanwhile, the amount of carbon sequestered per unit sown area in Guangdong's farmland ecosystems was generally flat (Fig. 3b), with an average annual carbon sequestration per unit sown area of 4.31 t ha^−1^. While the amount of carbon sequestered per unit arable land area showed an increasing trend, especially in 2017, when it started to rise rapidly, from 6.82 t ha^−1^ per unit arable land area in 2001 to 10.97 t ha^−1^ per unit arable land area in 2020, an increase of 60.85%. The average annual carbon sequestration per arable area is 7.25 t ha^−1^, an increase of 56.71% in the 4 years from 2017 to 2020.

#### Analysis of the role of crop carbon sequestrations in Guangdong's farmland ecosystems

**Figure 4:**
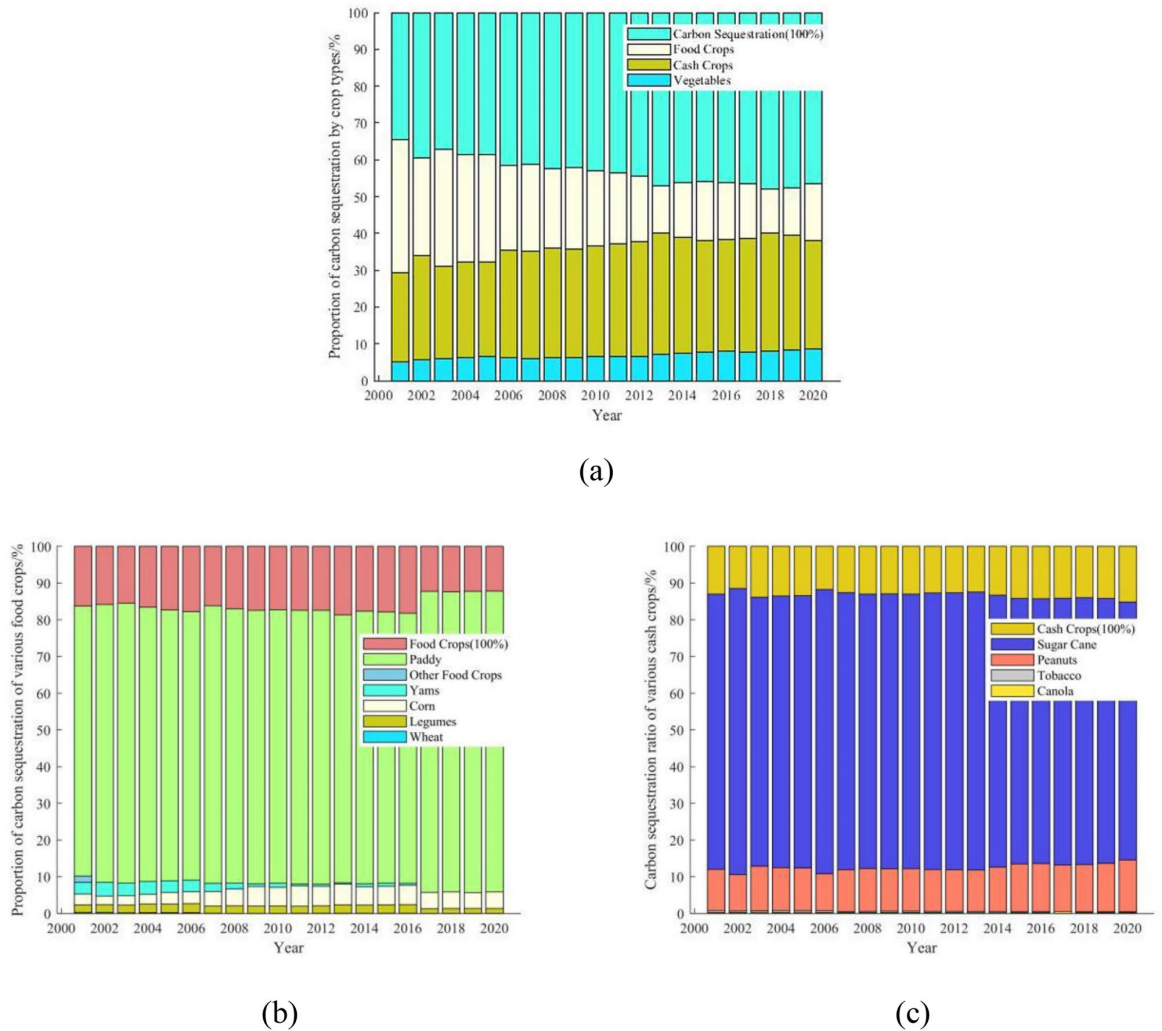
(**a**) Proportion of carbon sequestered by various crops in Guangdong farmland ecosystems. (**b**) Proportion of carbon sequestered by various food crops in Guangdong farmland ecosystems; (**c**) proportion of carbon sequestered by various cash crops in Guangdong farmland ecosystems.

As can be seen from Fig. 4a, food crops play the largest role in carbon sequestration in Guangdong's farmland ecosystems, with an average share of 56.95% of the total carbon sequestration in the past 20 years. Its share tends to decline over time, but the amount of carbon sequestered by food crops in Guangdong still reaches 10.209 million t a^−1^ in 2020. The carbon sequestration role of cash crops is next, rising from 29.43% in 2001 to 37.92% in 2020, with an average share of 36.17%, an increase of 28.85%, and average annual carbon sequestration of 6.8863 million t a^−1^. The inter-year variation of vegetables carbon sequestration also shows an increasing trend, rising from 5.02 to 8.73%, with an increase of 73.90%, and average annual carbon sequestration of 1.13112 million t a^−1^.

When the carbon sequestration capacity of food (Fig. 4b) and cash crops (Fig. 4c) in Guangdong's farmland ecosystems is broken down, it is easy to see that paddy is in an absolute position in terms of carbon sequestration among food crops, with an average share of 83.81% over the past 20 years and average annual carbon sequestration of 8.8946 million t a^−1^. Especially since 2017, the carbon sequestration share of paddy has risen to over 87% and will remain until 2020. Also, sugarcane's share of carbon sequestration in cash crops is absolute, with average annual share of 86.73% and an average annual carbon sequestration of 5.9712 million t a^−1^. while, peanut's share of carbon sequestration in cash crops is also not small, with average annual share of 12.47% and an average annual carbon sequestration of 0.8606 million t a^−1^.

**Figure 5:**
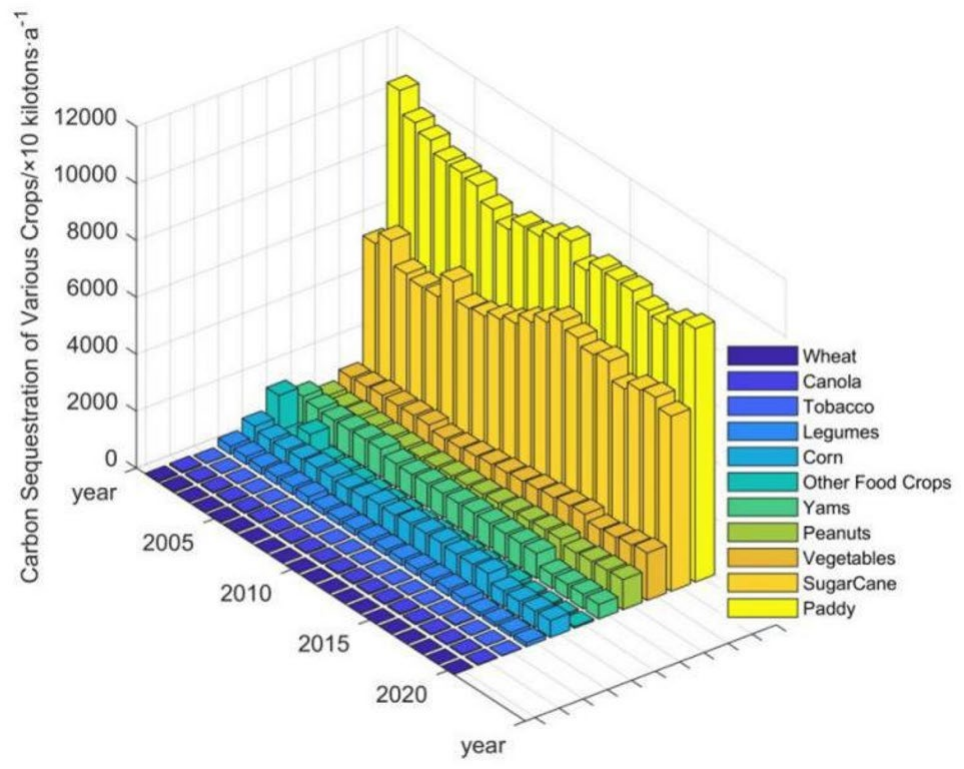
Comparison of carbon sequestration by various crops in Guangdong farmland ecosystems.

An analysis of the inter-annual variation in carbon sequestration of various crops (Fig. 5) shows that paddy and sugar cane play the largest role in carbon sequestration in Guangdong's farmland ecosystems. Their combined annual average carbon sequestration amounting to 14.8658 million t a^−1^, accounting for 78.09% of the total annual average carbon sequestration in Guangdong's farmland ecosystems. Vegetables, peanuts, and yams also play a significant role in carbon sequestration, with the combined annual average carbon sequestration of the three species being 2.9936 million t a^−1^, accounting for 15.73% of the total annual average carbon sequestration.

### Analysis of the carbon footprint of Guangdong's farmland ecosystems under the “dual carbon” target

**Figure 6:**
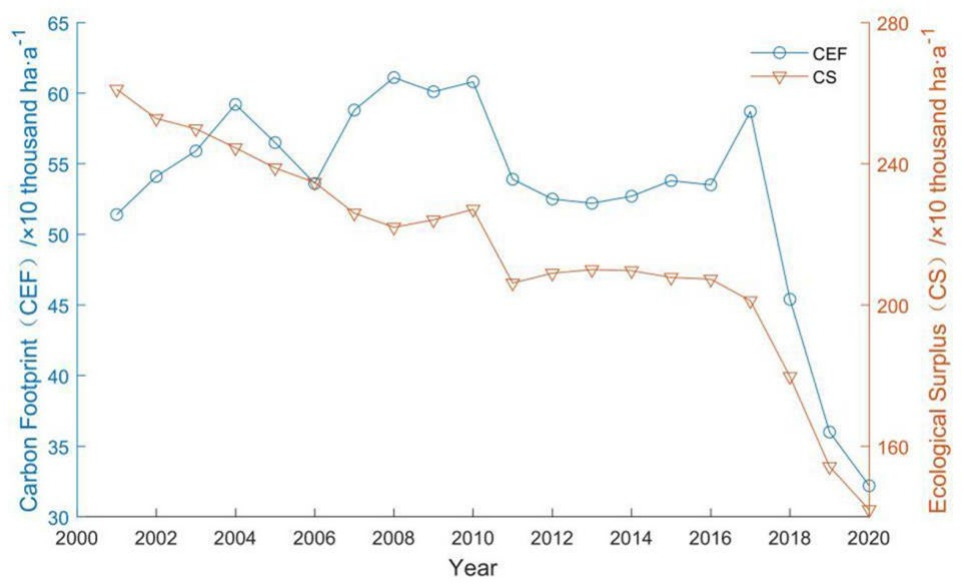
Inter-annual variation of carbon footprint and ecological surplus of farmland ecosystems in Guangdong.

The carbon footprint of Guangdong's farmland ecosystem ($CEF$) is 531,100 ha a^−1^ per year, showing a general decrease (Fig. 6), with a 59.65% decrease from 513,900 ha a^−1^ in 2001 to 321,900 ha a^−1^ in 2020. The carbon footprint of Guangdong's farmland ecosystems in the past 20 years (the peak value is 611,500 in 2008 ha a^−1^) is smaller than the ecological carrying capacity (i.e. the arable land area, the lowest value is 1.7421 million ha a^−1^ in 2020), and is in a state of carbon ecological surplus. Guangdong's farmland carbon surplus ($CS$) shows a decreasing trend year by year (Fig. 6), from 2.1611 million ha a^−1^ in 2001 to 1.4202 million ha a^−1^ in 2020, a decrease of 45.61%. Although the carbon footprint and the inter-annual variation of the carbon surplus both show a decreasing trend, the productive area required to absorb the carbon emissions from farmland (i.e. the carbon footprint) rises from 16.44 to 18.48% of the arable land area in the same period.

### An overview of the interannual variability of carbon emissions, sequestrations and footprints of farmland ecosystems in Guangdong

In the above analysis of the inter-annual variation of carbon emissions, sequestration, and footprint of Guangdong's farmland ecosystems, it was found that 2017 was a special year. After 2017, which the total carbon emissions from Guangdong's farmland ecosystems and carbon emissions due to agricultural chemicals (Fig. 1a), carbon emissions per unit of arable land area and sown area (Fig. 1b), carbon sequestration per unit of arable land area (Fig. 3b) and carbon footprint and carbon surplus (Fig. 6) all show a large turnaround. Based on the analysis of the factors after 2017 in Table 3, it can be seen that the number of various fertilizers using is gradually decreasing after 2017, especially the number of nitrogen fertilizers decreased by 149,600 t a^−1^ in 2018 compared with the amount of the previous year, a decrease of 14.44% in a single year, and the carbon emission decreased by 316,600 t a^−1^. The arable land area in Guangdong is decreasing after 2017, from 2017 to 2019, it decreased by 697,800 ha, a decrease of 26.84%, but the total carbon sequestration still remains above 19 million t a^−1^ (Fig. 3a), and while the area of arable land in Guangdong is decreasing, the area sown is climbing. The ratio of sown area to arable land area is used as the number of tillage per unit of arable land area in the paper, and the number of tillage per unit of the arable land area rises from 1.63 ha ha^−1^ in 2017 to 2.56 ha ha^−1^ in 2020.

**Table 3:** Inter-annual variation of selected factors in Guangdong agro-ecosystems, 2017–2020.

Year	2017	2018	2019	2020
Total arable land area (× thousands ha)	259.97	225.08	190.19	174.21
Total sown area (× thousands ha)	422.75	427.94	435.74	445.18
Carbon emissions per unit of arable land area (t ha^−1^)	1.58	1.66	1.90	2.03
Carbon emissions per unit sown area (t ha^−1^)	1.03	1.15	1.21	1.26
Carbon sequestration per unit of arable land area (t ha^−1^)	7.00	8.21	10.04	10.97
Nitrogen fertilizer dosage (× 10 ktons)	103.6	88.64	86.27	84.11
Phosphate fertilizer dosage (× 10 ktons)	24.80	27.05	26.45	25.80
Potassium fertilizer dosage (× 10 ktons)	50.70	44.86	43.27	41.68
Compound fertilizer dosage (× 10 ktons)	79.20	70.77	69.80	68.22

Based on the conclusions obtained, the author looked up the agriculture-related policies of Guangdong Province in 2016 and 2017. And found that on 30 December 2016, the Guangdong Provincial People's Government, in response to the soil prevention and control plan of the Central Government, formulated and issued to the cities and counties under its jurisdiction the Implementation Plan of the Guangdong Provincial Soil Pollution Prevention and Control Action Plan (here in after referred to as the “Plan”). The Plan encourages farmers in all areas to reduce the number of chemical fertilizers and apply pesticides scientifically. The effectiveness of the implementation of the Plan in Guangdong Province is remarkable as seen through the changes in the application of various fertilizers, which in the aspect of reducing fertilizer application alone resulted in a 344,900 t ha^−1^ reduction in carbon emissions from fertilizer inputs in 2018 compared to 2017. At the same time, the number of farmland tillage has increased, and the area of arable land has been reduced, but the total sown area of crops has remained relatively constant. In 2019, while the area of arable land in Guangdong (actual data on arable land in 2020 is missing, and the forecast alone may cause too much error, so 2019 is used as an example) is 69.78 ha less than that in 2017, the total sown area has increased by 22.43 ha, and the total agricultural output value still increased by RMB 64 billion. Which shows that the utilization rate of arable land and the output value per unit of arable land in Guangdong have both increased.

## Conclusion

The paper estimate carbon emissions, carbon sequestration, and carbon footprint of farmland ecosystems in Guangdong Province for the past 20 years from 2001 to 2020, and analyse the inter-annual variation. In calculating carbon emissions from farmland ecosystems, the carbon emission factors for nitrogen (2116 kgC t^−1^), phosphate (636 kgC t^−1^), and potash (180 kgC t^−1^) fertilizers were calculated using the carbon emission factors derived by Chen et al.^24^ for Chinese chemical fertilizers, which are more in line with the actual situation of nitrogen, phosphate, and potash fertilizers in China. The carbon emission factor for irrigation (20.476 kgC t^−1^) was also calculated by substituting the carbon emission correction factor derived from the study by Tian et al.^23^ on the current situation of irrigation in China. The carbon footprint of Guangdong Province in 2009 is 21.14% (i.e. 0.2114 ha ha^−1^), which is similar to the result (i.e. 0.20 ha ha^−1^) of Duan et al.^15^, who adopted the uncorrected carbon emission factor of fertilizer (895.6 kgC t^−1^) before subdivision to study the carbon footprint of Guangdong farmland ecosystem in 2009. Therefore, the results of the paper on the carbon footprint of Guangdong farmland ecosystems are credible and more consistent with the current situation of carbon emissions and carbon sequestration in Guangdong farmland ecosystems.

## Discussion

The total carbon emissions from Guangdong's farmland ecosystems first showed an increasing trend year by year, and then turned to a decreasing trend year by year after 2017, with its annual average carbon emissions per unit of the arable land area and per unit of the sown area being 1.43 t ha^−1^ and 1.19 t ha^−1^ respectively. Among them, the carbon emissions from agricultural chemicals contributed the most, accounting for 85.51% of the annual average, and its inter-annual variation trend was consistent with that of the total carbon emissions, with the overall change is relatively flat. Fertilizers account for the largest share of carbon emissions from agricultural chemicals, followed by pesticides. The proportion of carbon emissions from compound fertilizers, phosphate fertilizers, and potash fertilizers to total carbon emissions from fertilizers is generally on the rise, and the total carbon emissions from all three are generally on the rise, with carbon emissions from compound fertilizers and phosphate fertilizers reaching a peak in 2016.

The carbon sequestration function of Guangdong's farmland ecosystems was generally on a declining trend, with a turnaround after 2017 and an increasing trend year by year. The average annual carbon sequestration per unit of the arable land area and per unit of the sown area were 7.25 t ha^−1^ and 4.31 t ha^−1^ respectively, with the carbon sequestration per unit of the arable land area showing a rapidly increasing trend after 2017. Food crops have the largest carbon sequestration function, followed by cash crops. Paddy and sugarcane were the crops with the largest carbon sequestration function among food crops and cash crops, respectively, and the sum of their annual average carbon sequestration accounted for 78.09% of the total carbon sequestration in Guangdong's farmland ecosystems.

The carbon footprint of Guangdong's farmland ecosystems from 2001 to 2020 shows an overall decreasing trend, with an average carbon footprint of 531,100 ha a^−1^ and in a carbon ecological surplus. But the carbon surplus is decreasing year by year, and the proportion of carbon footprint to the arable land area in the same period is increasing year by year.

Guangdong's total carbon emissions from farmland ecosystems and agricultural chemical emissions turned around after 2017, changing from a year-on-year increase to a decline. Carbon emissions and sequestration per unit of the arable land area and carbon emissions per unit of the sown area turned to a year-on-year increase after 2017, and the carbon footprint experienced a sudden drop after 2017.

According to the findings of the paper, Guangdong's farmland ecosystem has achieved good results since the implementation of the soil control program in 2017, with the land-use efficiency has improved, and a significant improvement in fertilizer application efficiency, and still a steady increase in agricultural output. Therefore, other provinces in China can refer to Guangdong's initiatives in developing policies to reduce emissions from farmland under the context of the “dual carbon” target. The carbon surplus from Guangdong's farmland ecosystems can be used to compensate for other industries in the industrial restructuring under the context of the “dual carbon” target, which can serve the long-term goal of “carbon neutrality”. However, the carbon surplus of the farmland ecosystem is decreasing year by year, and the carbon compensation capacity provided by agriculture is weakening year by year, which is related to the reduction of the cultivated land area. It is necessary to be more stringent and serious in the formulation and implementation of the policy on the protection of the cultivated land area, and give full play to the carbon sequestration capacity of the farmland ecosystem. Carbon emissions from Guangdong's farmland ecosystems are mainly influenced by fertilizer application. If the government want to reduce carbon emissions, it can start with the structure and efficiency of fertilizer application and reduce the reliance on nitrogen fertilizer.

The Guangdong government should also consider the turnover of soil organic carbon carefully while formulating policies to reduce ineffective arable land use and increase the frequency of tillage. It can also refer to the research results of the paper and relevant researchers, such as increasing organic fertilizer application measures to reduce soil organic carbon loss^25^ and taking the opportunity to improve the relevant organic fertilizer industry chain to further reduce carbon emissions.

## Limitation

About 10% of the Earth's organic carbon is stored in agricultural soils^26^, and the increase in soil organic carbon in farmland ecosystems is thought to play an important role in offsetting anthropogenic carbon emissions and mitigating climate change^27,28^. However, soil organic carbon in agroecosystems is affected by factors such as fertilizer application^29,30^, land tillage^31,32^, and climate^33^, which affect the turnover rate of soil organic carbon. In the paper, the carbon footprint accounting was carried out using traditional methods, which did not consider the changes in soil organic carbon loss due to climate warming, changes in the structure of fertilizer application, and increased land tillage. Therefore, subsequent studies can address the above limitations by conducting research on the carbon sequestration rate and carbon respiration rate of different agricultural soils.

## Data Availability

The paper dates are obtained from the Guangdong Provincial Statistical Yearbook, the China Statistical Yearbook, and the China Rural Statistical Yearbook from 2002 to 2021. When the data in the provincial yearbooks differ from the national yearbooks, as well as the data in the preceding and following years, the China Statistical Yearbook, and the latest yearbook data prevail. The interpolation method and ARIMA forecasting model are used in the paper^24^, and the missing arable land area data of Guangdong Province in 2018 and 2020 are predicted. The datasets generated and analysed during the current study are available in the FIGSHARE repository, 10.6084/m9.figshare.19336937. It includes raw data on indicators such as various types of fertilizers, agricultural films, agricultural machinery power and various types of grain production in Guangdong Province from 2001 to 2020, as well as the calculation results of carbon emissions and sequestration for various types of agricultural production inputs and outputs.

## References

[1] Guan Y, Shan Y, Huang Q (2021). Assessment to China's recent emission pattern shifts. Earth's Future.

[2] Liu F, Tang L, Liao K (2021). Spatial distribution and regional difference of carbon emissions efficiency of industrial energy in China. Sci. Rep..

[3] Raich JW, Tufekciogul A (2000). Vegetation and soil respiration: Correlations and controls. Biogeochemistry.

[4] Black CC (1973). Photosynthetic carbon fixation in relation to net CO_2_ uptake. Annu. Rev. Plant Physiol..

[5] Wackernagel M, Rees W (1998). Our Ecological Footprint: Reducing Human Impact on the Earth.

[6] East AJ (2008). What is a Carbon Footprint? An Overview of Definitions and Methodologies.

[7] Liu XH, Xu WX, Li ZJ (2013). The missteps, improvement and application of carbon footprint methodology in farmland ecosystems with the case study of analyzing the carbon efficiency of China's intensive farming. Chin. J. Agric. Resour. Reg. Plan.

[8] Zhao, R., Qin, M. Temporospatial variation of partial carbon source/sink of farmland ecosystem in Coastal China. *J. Ecol. Rural Environ*. **2** (2007).

[9] Zhao RQ, Liu Y, Ding ML (2010). Research on carbon source and sink of farmland ecosystem in Henan Province. J. Henan Agric. Sci..

[10] Xiao-yong, Q. Spatial-temporal variation and impact factor of carbon source and sink of farmland ecosystem in Shanghai, China. *J. Agro-Environ. Sci*. **07** (2011).

[11] Liang W, Jie Z, Shouyue C (2016). Analysis of ecosystem carbon sources/sinks and carbon footprint in farmland ecosystem of Shandong Province. J. China Agric. Univ..

[12] Dong G, Mao X, Zhou J (2013). Carbon footprint accounting and dynamics and the driving forces of agricultural production in Zhejiang Province, China. Ecol. Econ..

[13] Xu X, Zhang B, Liu Y (2013). Carbon footprints of rice production in five typical rice districts in China. Acta Ecol. Sin..

[14] She W, Wu Y, Huang H (2017). Integrative analysis of carbon structure and carbon sink function for major crop production in China's typical agriculture regions in China. J. Clean. Prod..

[15] Duan HP, Zhang Y, Zhao JB (2011). Carbon footprint analysis of farmland ecosystems in China. Soil Water Conserv.

[16] Han ZY, Meng YL, Xu J (2012). Temporal and spatial difference in carbon footprint of regional farmland ecosystem-taking Jiangsu Province as a Case. J. Agro-Environ. Sci..

[17] Gan Y, Liang C, Chai Q (2014). Improving farming practices reduces the carbon footprint of spring wheat production. Nat. Commun..

[18] Liu C, Cutforth H, Chai Q (2016). Farming tactics to reduce the carbon footprint of crop cultivation in semiarid areas. A review. Agron. Sustain. Develop..

[19] Hao M, Hu H, Liu Z (2019). Shifts in microbial community and carbon sequestration in farmland soil under long-term conservation tillage and straw returning. Appl. Soil. Ecol..

[20] Mohammed, S., Mirzaei, M., Pappné Törő, Á., *et al*. Soil carbon dioxide emissions from maize (*Zea mays* L.) fields as influenced by tillage management and climate. *Irrig. Drainage*. (2021).

[21] West TO, Marland G (2002). A synthesis of carbon sequestration, carbon emissions, and net carbon flux in agriculture: comparing tillage practices in the United States. Agric. Ecosyst. Environ..

[22] Tian, Y., Li, B., Zhang, J. Research on stage characteristics and factor decomposition of agricultural land carbon emission in China. *J. China Univ. Geosci. (Social Sci. Edn.)*. **1** (2011).

[23] Chen S, Lu F, Wang XK (2015). Estimation of greenhouse gases emission factors for China's nitrogen, phosphate, and potash fertilizers. Acta Ecol. Sin..

[24] Zaho M, Liu WZ (2015). Comparison of short-term cotton price forecasted by ARIMA model and smooth ARIMA model in China. Guizhou Agric. Sci..

[25] Yang ZC, Zhao N, Huang F (2015). Long-term effects of different organic and inorganic fertilizer treatments on soil organic carbon sequestration and crop yields on the North China Plain. Soil Tillage Res..

[26] Liang F, Li J, Yang X (2016). Three-decade long fertilization-induced soil organic carbon sequestration depends on edaphic characteristics in six typical croplands. Sci. Rep..

[27] Lal R (2004). Agricultural activities and the global carbon cycle. Nutr. Cycl. Agroecosyst..

[28] Lal R (2004). Soil carbon sequestration to mitigate climate change. Geoderma.

[29] Kaur T, Brar BS, Dhillon NS (2008). Soil organic matter dynamics as affected by long-term use of organic and inorganic fertilizers under maize-wheat wheat cropping system. Nutr. Cycl. Agroecosyst..

[30] Khan SA, Mulvaney RL, Ellsworth TR (2007). The myth of nitrogen fertilization for soil carbon sequestration. J. Environ. Qual..

[31] Baker JM, Ochsner TE, Venterea RT (2007). Tillage and soil carbon sequestration—What do we really know?. Agric. Ecosyst. Environ..

[32] Yi-xiang W, Zhi-dan WU, Bo-qi W (2010). Effects of tillage and grass intercropping on soil respiration in citrus reticulate orchard. Chin. J. Agrometeorol..

[33] Chen S, Wang W, Xu W (2018). Plant diversity enhances productivity and soil carbon storage. Proc. Natl. Acad. Sci..

